# Validation of the internal structure of the Brazilian Dental Vulnerability Scale

**DOI:** 10.11606/s1518-8787.2023057005360

**Published:** 2024-04-01

**Authors:** Daniele Boina de Oliveira, Flávio Rebustini, Danielle da Costa Palacio, Marcio Cardozo Paresque, Ilana Eshriqui Oliveira, Wander Barbieri, Danielle Viana Ribeiro, Debora Heller, Daiana Bomfim, Tamara Kerber Tedesco

**Affiliations:** I Universidade Cruzeiro do Sul Departamento de Odontologia São Paulo SP Brasil Universidade Cruzeiro do Sul. Departamento de Odontologia. São Paulo, SP, Brasil; II Universidade de São Paulo Escola de Ciências, Humanidades e Artes Programa de Pós-graduação em Gerontologia São Paulo SP Brasil Universidade de São Paulo. Escola de Ciências, Humanidades e Artes. Programa de Pós-graduação em Gerontologia. São Paulo, SP, Brasil; III Hospital Israelita Albert Einstein Centro de Estudos, Pesquisa e Prática em Atenção Primária à Saúde e Redes Diretoria de Atenção Primária à Saúde e Rede Assistencial São Paulo SP Brasil Hospital Israelita Albert Einstein. Centro de Estudos, Pesquisa e Prática em Atenção Primária à Saúde e Redes. Diretoria de Atenção Primária à Saúde e Rede Assistencial. São Paulo, SP, Brasil; IV Universidade de São Paulo Faculdade de Odontologia Departamento de Ortodontia e Odontopediatria São Paulo SP Brasil Universidade de São Paulo. Faculdade de Odontologia. Departamento de Ortodontia e Odontopediatria. São Paulo, SP, Brasil

**Keywords:** Oral Health, Primary Health Care, Population Health Management

## Abstract

**OBJECTIVE:**

This study aimed to evaluate evidence of validity of internal structure of the Brazilian Dental Vulnerability Scale (EVO-BR) when applied in Brazil.

**METHODS:**

This is a psychometric study that seeks to validate a scale elaborated by evidence of internal structure. Data collection was conducted in 18 basic health units that implement the Brazilian Healthcare Planning (PAS) methodology, across the five regions of Brazil. The initial version of the EVO-BR contained 41 items that measured dental vulnerability and was applied to users of the Brazilian Unified Health System (SUS) aged 18 years or older who were in basic health units for consultation with higher education professionals. To evaluate the evidence, the following statistical analyses were performed: exploratory factor analysis, confirmatory factor analysis, and network analysis.

**RESULTS:**

A total of 1,753 users participated in the study. To adjust the sample, we considered the factorability obtained from Kaiser-Meyer-Olkin (KMO) test = 0.65, Bartlett sphericity test = 8019.7, and a matrix determinant of 0.008. The initial parallel analysis indicated a four-dimensional model and had the items adjusted according to factor loading (ranging from 0.38 to 0.99), common factors (0.13 to 0.89), and Pratt’s measure, until the model presented congruence in the statistical and interpretative principles simultaneously. The final model contained 15 items, maintaining the four dimensions indicated by the parallel analysis, and held an explained variance of 68.56%.

**CONCLUSIONS:**

The EVO-BR is a validated scale to measure dental vulnerability and, thus, can contribute to the organization of access to the oral health team in primary health care (PHC) by stratifying the population, as recommended in the Brazilian Healthcare Planning.

## INTRODUCTION

The challenge of ensuring access to oral health in a universal and equitable manner remains in the Brazilian Unified Health System (SUS). In this context, authors have been discussing formats to organize supply and demand by population-based management. Strategies such as the Brazilian Healthcare Planning (PAS) aim to overcome historical fragmentation and the supply base management model^1^.

The PAS acts in the micro and macro processes of primary health care (PHC) and is based on the knowledge of the subpopulations that compose the territory, so that care can be organized considering their needs. This requires tools that can support professionals in these processes.

Vulnerability is the result of multidimensional and relational conditions, and is determined by a set of individual, social, and programmatic factors. In oral health, dental vulnerability has recently been defined as “a set of factors of the social, structural, and general, mental, oral health dimensions, in addition to health services and public management that influence the dynamics of the health-disease process in dentistry.”^2^ In light of this multifactorial context and the knowledge gap in oral healthcare, an instrument that allows the identification of dental vulnerability in a systematic and standardized manner across Brazil is essential to support the planning of oral health actions in a timely and equitable manner.

In this sense, the Dental Vulnerability Scale (EVO) was developed and demonstrated evidence of validity in a study conducted in the city of São Paulo, lacking validity in a national context. Considering the diversity and extension of the Brazilian territory, it is necessary to investigate the evidence of validity of EVO at the national level. Thus, this study aims to investigate the evidence of efficacy of the internal structure of the Brazilian Dental Vulnerability Scale (EVO-BR) in PHC, in different Brazilian contexts. EVO-BR is believed to support the planning of oral health policies that expand access to healthcare services based on the measurement of dental vulnerability, reducing access inequality in Brazil.

## METHODS

This study aims to provide additional evidence on the internal structure of the EVO-BR by analyzing it using exploratory factor analysis (EFA), confirmatory factor analysis (CFA), and network analysis (NA), following current recommendations from the American Educational Research Association, the American Psychological Association, and the US National Council on Measurement in Education. The study was approved by the Research Ethics Committee of the Hospital Israelita Albert Einstein (CAAE No. 12395919.0.0000.0071).

### Study Location

The sample was composed of users of basic health units (BHUs) located in the five Brazilian geographic regions. BHUs were selected according to the following criteria: located in municipalities that implement the PAS methodology, considering that at least one BHU was selected from each of the five Brazilian geographic regions; located in the most populous municipalities; and with easy access to data collectors.

Based on these criteria, EVO-BR was applied in the municipalities of: Uberlândia-MG (two BHUs) and São Paulo-SP (11 BHUs), covering the Southeast; Irati and Teixeira Soares-PR (two BHUs), covering the South; Belo Jardim-PE (one BHU), covering the Northeast; Rondonópolis-MT (two BHUs), covering the Midwest; and, finally, Boa Vista-RR (one BHU), covering the North. Data collection was conducted in two stages, the first from September to November 2019 (SP) and the second from May to August 2022 (MG, MT, RR, PE, PR).

The first stage of collection was performed by dentists^3^ and the second by trained data collectors, during a two-week visit to the municipalities outside São Paulo.

### Sample

Inclusion criteria were users of the service; aged 18 years or older; and attending a BHU during the data collection period. Users with lack of information about the EVO items (i.e., those who agreed to participate and answered the characterization instrument but had data collection interrupted before answering the items predicted in the scale) were excluded.

Participants were approached during care at the dental clinic or in the waiting room of the BHU and invited to participate in the research. After signing the informed consent form, the clinical and sociodemographic characterization structured questionnaire and the EVO-BR items were applied (Table 1)^3^. Research Electronic Data Capture (RedCap) software program was used for data collection and storage.

**Table 1:** Validation of the Dental Vulnerability Scale.

Item	Yes	No
1. Are you a bedridden patient?		
2. Do you live only indoors?		
3. Do you have the HIV virus?		
4. Do you have heart disease?		
5. Does your health prevent you from doing some daily activities?		
6. Do you eat foods that contain added sugar every day?		
7. Do you eat pasta, potatoes, or breads in every meal?		
8. Do you have diabetes?		
9. Do you have any movement difficulties?		
10. Do you have any illnesses that need follow-up?		
11. Do you have Down syndrome?		
12. Do you smoke?		
13. Do you have cancer?		
14. Have you ever undergone radiation therapy?		
15. Do you consume alcoholic beverages?		
16. Do you use drugs?		
17. Do you consider your family to have good hygiene habits?		
18. Do you have any missing teeth?		
19. Do you believe that tooth problems are better solved with extraction?		
20. Do you consider yourself to have good oral health?		
21. Do you have mouth cancer?		
22. Have you ever had mouth cancer?		
23. Do you have decayed teeth?		
24. Do you consider it important to take care of your mouth?		
25. Do you believe that diseases of the mouth can be prevented?		
26. Do you consider yourself responsible for the health of your mouth?		
27. Do you consider it important to have all your teeth in your mouth?		
28. Do you chew well?		
29. Do you have a diagnosis of depression?		
30. Do you have access to fluoride water?		
31. Do you have a bathroom in your home?		
32. Do you have electricity in your home?		
33. Do you have running water in your home?		
34. Is there a sewage collection system in your home?		
35. Do you consider your home far from the dental care service you use?		
36. Do you know the public health center where you can visit?		
37. Do you visit public health centers?		
38. Are you followed up by an oral health team?		
39. Do you visit the dentist for free?		
40. Have you ever been to the dentist for treatment?		
41. Do you have a dental insurance plan?		

### Statistical analysis

**Exploratory factor analysis (EFA):** the verification of data is fundamental, and they are susceptible to factor analysis via the measure of sampling adequacy. For this stage, Bartlett sphericity, matrix determinant, and KMO were measured. In addition to assessing the database, an individual analysis of items was conducted, as recommended, so that their unsuitability for factoring could affect the solution of the model. The missing values were treated using the multiple imputation technique^3^.

Dimensionality was tested by the optimal implementation of parallel analysis (PA) with minimum rank factor analysis, which minimizes the common variance of residuals. PA has been implemented using polychoric matrix and random permutation of 500 matrices. This strategy has been considered one of the most robust and accurate techniques for dimensionality testing^4,5^. The factor extraction was performed by the unweighted least squares (ULS) technique, which reduces the matrix residues. If the instrument showed multidimensionality, the Promin oblique rotation was used^6^. The following indicators were adopted to evaluate unidimensionality: unidimensional congruence (UNICO) > 0.95, explained common variance (ECV) > 0.80, and mean of item residual absolute loadings (MIREAL) < 0.30^7^.

**Instrument quality parameters:** the explained variance of the instrument should be around 60%^8^. Initial factor loadings of 0.30 are recommended when the sample comprises 300 individuals at least. The permanence or removal of the item in the model will depend on the magnitude of the factor loadings, common factors, absence of double saturation, Heywood cases, and interpretability of factors. To increase the accuracy of decision-making regarding the permanence or removal of items, the unique directional correlation coefficient (ETA) by Pratt’s measure was used^9^.

**Confirmatory factor analysis (CFA):** for the primary CFA data, the factor loadings and predictive power of the item (R^2^) were used. The model’s fit indices were: χ^2^/df; Non-Normed Fit Index (NNFI) > 0.95; Comparative Fit Index (CFI) > 0.95; Goodness Fit Index (GFI) > 0.95; Root Mean Square Error of Approximation (RMSEA) < 0.08; and Root Mean Square of Residuals (RMSR) < 0.8. The model tested in CFA was the factorial solution found in the initial EFA study^2^.

Reliability was measured by four indicators: Cronbach’s alpha^10^, Greatest Lower Bound^11^, Omega^12^— these three by Bayesian estimation — and Overall Reliability of Fully-Informative Prior Oblique N-EAP scores (ORION)^13^.

**Network analysis:** over the last decade, network analysis has been extended to various scenarios, such as symptom assessment^14^, psychological networks and psychopathologies, post-traumatic stress^15^, anxiety^16^, the development of measurement instruments^17^, and dentistry^18^.

It is important to understand how network analysis can be useful in the search for evidence of validity. According to Newman^19^, the analysis consists of two stages: in the first, a statistical model of the data is estimated, from which some parameters can be represented as a weighted network between the observed variables, and in the second stage, the structure of the weighted network is analyzed using measures derived from graph theory.

This study used the High-dimensional Undirected Graph Estimation (HUGE)^20^ technique as estimator and the Extended Bayesian Information Criterion (EBIC) as criterion. Huge works with two estimation procedures: 1) neighborhood search algorithm; and 2) Lasso graph algorithm^21^. The graph nodes were positioned using the Fruchterman-Reingold algorithm^22^, which is based on the strength and connectivity between nodes, each one representing an item of the instrument.

Four indicators were used to evaluate the model: betweenness, which assesses the efficiency with which a node connects to others; closeness, which assesses how easily information reaches other nodes from a specific node; strength or degree, which represents how connected a node is to the rest of the network; and finally, expected influence, which aims to assess the nature and strength of a node’s cumulative influence within the network^23^ and thus the role it can be expected to play in its activation, persistence, and remission^24^.

The literature has recommended adopting multiple tests and techniques to adjust an instrument, meeting contemporary recommendations for evidence of validity. This combination aims to improve the accuracy and quality of the instruments^25,26^ and can help determine which model is best when there is more than one possible solution^27^. For both techniques, a bootstrap of 5000 was applied. The analyses were performed as Factor 12.02 and JASP 16.04.

## RESULTS

The study population comprised 1,753 participants. Most respondents were male (52.01%), with a mean age of 39 years (14.37%). Regarding ethnicity/skin color, the distribution was similar between White (30.20%) and Black and Mixed-race (32.69%), with a predominance of Black individuals (34.44%). Most people (81.42%) reported that the ratio of residents/rooms in the household was equal to or less than one.

The analyses were performed with the initial 41 items of the EVO-BR (Table 1). The measure of sample adequacy indicated the possibility of factorability with KMO = 0.65; Bartlett sphericity = 8019.7 (df = 820; p < 0.0001); and matrix determinant of 0.008.

The initial PA indicated a four-dimensional model, but with several items with inadequate levels of factor loading, common factors, and Pratt’s measure. This led to the successive removal of items to adjust the statistical and interpretative principles of the model until they became congruent. The final model contained 15 items, maintaining the four dimensions indicated by the parallel analysis, and held an explained variance of 68.56%. The closeness of dimensionality values indicated a multidimensional model: UNICO = 0.79; ECV = 0.63; and MIREAL = 0.21. Table 2 presents the final model with factor loadings, common factors, and Pratt’s measure. The factor loadings of the instrument ranged from 0.38 to 0.99, common factors from 0.13 to 0.89, and the ETA of the Pratt’s measure from 0.36 to 0.94

**Table 2:** Factor loadings, common factors, and Pratt’s measure.

Item		Factorial loadings				h^2^	Pratt´s measure (ETA)			
	
		General Health	Oral Health	Infrastructure	Health Services		General Health	Oral Health	Infrastructure	Health Services
V1	Does your health prevent you from doing some daily activities?	**0.946**	0.011	-0.003	0.000	0.894	**0.945**	0.011	0.000	0.000
V2	Do you have any movement difficulties?	**0.831**	-0.049	-0.002	0.044	0.696	**0.832**	0.046	0.000	0.053
V3	Do you have any illnesses that need follow-up?	**0.680**	0.068	-0.064	-0.037	0.466	**0.678**	0.054	0.045	0.029
V4	Do you consider it important to take care of your mouth?	-0.061	**0.548**	-0.012	0.179	0.356	0.057	**0.556**	0.000	0.209
V5	Do you believe that diseases of the mouth can be prevented?	0.069	**0.467**	0.082	0.034	0.262	0.071	**0.483**	0.143	0.064
V6	Do you consider yourself responsible for the health of your mouth?	-0.118	**0.522**	0.005	0.087	0.307	0.116	**0.528**	0.033	0.115
V7	Do you consider it important to have all your teeth in your mouth?	0.019	**0.993**	-0.011	-0.073	0.965	0.018	**0.982**	0.000	0.000
V8	Do you have a bathroom in your home?	-0.079	0.201	**0.857**	0.138	0.982	0.063	0.319	**0.907**	0.231
V9	Do you have electricity in your home?	-0.034	-0.007	**0.647**	0.002	0.417	0.023	0	**0.645**	0.020
V10	Do you have running water in your home?	0.044	-0.043	**0.932**	0.042	0.87	0.056	0	**0.925**	0.109
V11	Is there a sewage collection system in your home?	0.009	-0.127	**0.861**	-0.164	0.643	0.016	0	**0.801**	0.000
V12	Do you know the public health center where you can visit?	0.039	0.211	0.221	**0.510**	0.475	0.049	0.273	0.307	**0.552**
V13	Do you visit public health centers?	0.136	0.07	0.179	**0.761**	0.737	0.151	0.127	0.269	**0.791**
V14	Are you followed up by an oral health team?	-0.054	-0.01	-0.062	**0.382**	0.139	0.048	0.000	0.000	**0.369**
V15	Do you visit the dentist for free?	-0.079	-0.142	-0.13	**0.831**	0.655	0.066	0.104	0.000	**0.800**

**Bold** - significant factor loadings (> 0.30).

ETA: Unique Directional Correlation.

The first dimension was called “General Health” and consisted of Items 1 to 3; the second dimension was called “Oral Health” with Items 4 to 7; the third dimension was called “Infrastructure” with Items 8 to 11; and the last dimension was called “Health Services” with Items 12 to 15. The model was identical to the initial model^3^.

The reliability indices with Bayesian estimation showed Cronbach’s alpha = 0.81 (95%CI 0.79-0.82), McDonald’s omega = 0.78 (95%CI 0.76-0.81), and GLB = 0.93 (95%CI 0.92-0.93). The ORION was, respectively, for each dimension, 0.95, 0.84, 0.92, and 0.98. All indicators are at appropriate levels.

**Figure 1:**
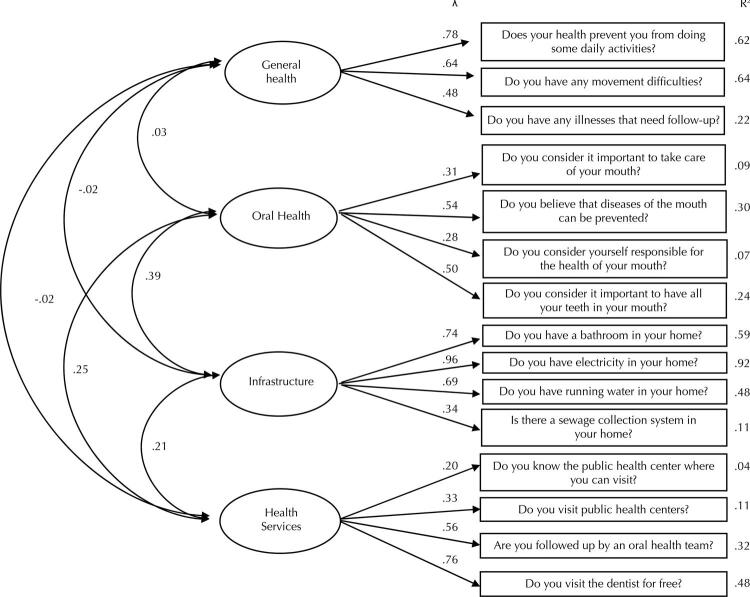
Path diagram.

The model obtained in the EFA was replicated in the CFA, with factor loadings ranging from 0.20 to 0.96 and the predictive capacity of the item (R2) from 0.09 to 0.92 (Figure 1). “General Health” had factor loadings ranging from 0.48 to 0.78; “Oral Health” from 0.28 to 0.54; “Infrastructure” from 0.34 to 0.96; and “Health Services” from 0.20 to 0.76. In addition to the primary indicators, the quality indices of the model were established as: X^2^_(51)_ = 2.96, p < 0.001; NNFI = 0.98; CFI = 0.99; GFI = 0.98; RMSEA = 0.03; and RMSR = 0.04. The indicators were established at satisfactory and consistent levels.

**Figure 2:**
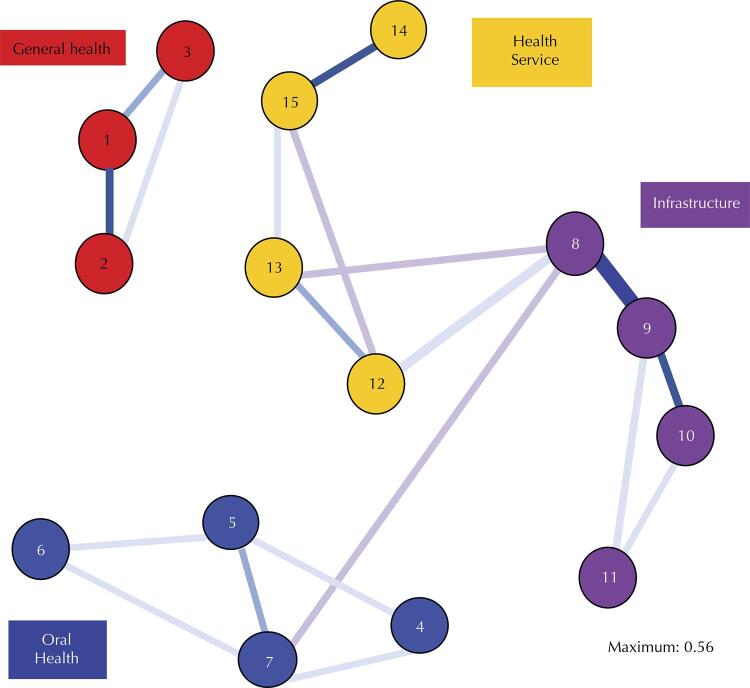
Network analysis of EVO-BR.

**Figure 3:**
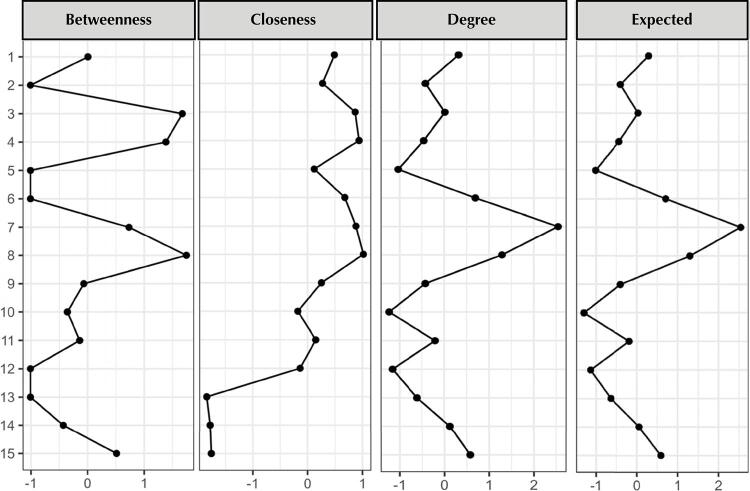
Centrality indices of the standardized items (z).

Network analysis was applied to the previously established model (Figure 2). Figure 3 shows that the items were appropriately associated with their correlated pairs, respecting the established domains. This indicates, again, the sustainability and stability of the proposed model.

EVO-BR: Validation of the Dental Vulnerability Scale

For the standardized centrality indicators, the items (Figure 3) that showed the most relevant results were: for betweenness, Items 3 “Do you have any illnesses that need follow-up?” and 8 “Do you have a bathroom in your house?”. For closeness the most relevant results were present in items that allow the flow of information in a shorter space, specifically in Items 3 “Do you have any illness that needs follow-up?”, 4 “Do you consider it important to take care of your mouth?”, 6 “Do you consider yourself responsible for the health of your mouth?”, 7 “Do you consider it important to have all your teeth in your mouth?”, and 8 “Do you have a bathroom in your home?”. For the strength/degree index and expected influence, Item 7 “Do you consider it important to have all your teeth in your mouth?” showed the best result, indicating the highest connection strength and the greatest cumulative influence for the model’s configuration.

## DISCUSSION

This study demonstrated, by using the initial model in the EFA with a multicenter sample and the replication of this model in the CFA and network analysis, that the EVO-BR is consistent, robust, and reliable in Brazil. In addition to suitable psychometric properties, the final version of the scale is a practical, concise, and easily applicable instrument since it comprises only 15 items, all with yes or no response options. Thus, the EVO-BR minimizes the subjectivity of responses and allows an accurate measurement of dental vulnerability by various primary healthcare professionals, including community health agents, not limited to dental professionals.

Despite this study being conducted with a convenience sample, not statistically representative of the Brazilian population, a notable strength is its inclusion of users from all five geographic regions of Brazil, thus encompassing the country’s territorial, cultural, and social diversity.

Corroborating the study conducted in São Paulo^3^, EVO-BR maintains the dimensions of general health, oral health, infrastructure, and health services to allow measurement of dental vulnerability. The general and oral health exhibit a strong correlation in the context of the health-disease process and impact the patient’s quality of life, becoming inseparable^28^. This fact can be explained by the association of oral problems with nutritional imbalances, interference in sleep quality, speech, worsening of psychosocial conditions, among others.

According to Gonçalves et al.^29^, most BHUs in Brazil lack the necessary infrastructure to ensure universal access and accessibility to dental services, which highlights the importance of this dimension for at least 23.1% of Brazilians who have some form of limitation^29^. Poor oral health service infrastructure is associated with reduced use of dental services, which are considered fundamental for characterizing dental vulnerability

Historical factors inherent in the late integration of dentistry into the Brazilian Family Health Strategy have contributed to reduced attention to oral health by the population, as well as weakened management. It is estimated that about 13% of the Brazilian population has never been to the dentist^30^. The items of EVO-BR, in the oral health dimension, precisely encompass this self-perception approach that reflects individuals’ experiences and quality of life, portraying their needs. This supports the population management model and challenges the current model of care, characterized by invasive procedures and low effectiveness for years.

It is known that individuals with better living conditions are more likely to receive dental care^28^. In this context, the dimension of health services considers access to and use of services in the measurement of dental vulnerability.

The PAS methodology aims to support healthcare managers and professionals in organizing health services and care networks, using *Modelo de Atenção às Condições Crônicas*^1^ (MACC – Care Model for Chronic Conditions) as its reference framework. The MACC considers elements of the Chronic Care Model (CCM), the risk pyramid model, and the social determinants of health model. Therefore, it is suggested that work processes be organized based on an understanding of social factors, extending beyond the biopsychosocial risk factors^1^.

## CONCLUSION

The EVO-BR proved to be a potential instrument for use in the Brazilian Healthcare Planning (PAS), aiming to support the work process and the planning of oral health actions in PHC, based on the needs of users. This helps promote comprehensive, equitable, and timely care for patients. Based on the identification of vulnerable groups, the EVO-BR is a strategy that contributes to advancing the discussion on organizing oral health services in Brazil.
