# Supplementary material for: Validation of the internal structure of the Brazilian Dental Vulnerability Scale
Source: Rev Saude Publica. 2024 Apr 1;57(Suppl 3):8s. doi: 10.11606/s1518-8787.2023057005360 (PMC11037902; doi:10.11606/s1518-8787.2023057005360)
Supplement: Translation

# Translation: Validação da estrutura interna da Escala Brasileira de Vulnerabilidade Odontológica

**Authors:** Daniele Boina de Oliveira, Flávio Rebustini, Danielle da Costa Palacio, Marcio Cardozo Paresque, Ilana Eshriqui Oliveira, Wander Barbieri, Danielle Viana Ribeiro, Debora Heller, Daiana Bomfim, Tamara Kerber Tedesco

**Affiliations:** I Brasil Universidade Cruzeiro do Sul. Departamento de Odontologia. São Paulo, SP, Brasil; II Brasil Universidade de São Paulo. Escola de Ciências, Humanidades e Artes. Programa de Pós-graduação em Gerontologia. São Paulo, SP, Brasil; III Brasil Hospital Israelita Albert Einstein. Centro de Estudos, Pesquisa e Prática em Atenção Primária à Saúde e Redes. Diretoria de Atenção Primária à Saúde e Rede Assistencial. São Paulo, SP, Brasil; IV Brasil Universidade de São Paulo. Faculdade de Odontologia. Departamento de Ortodontia e Odontopediatria. São Paulo, SP, Brasil

**Keywords:** Saúde Bucal, Atenção Primária à Saúde, Gestão da Saúde da População

## Abstract

**OBJETIVO:**

Avaliar evidências de validade da estrutura interna da Escala Brasileira de Vulnerabilidade Odontológica (EVO-BR) quando aplicada no Brasil.

**MÉTODOS:**

Trata-se de um estudo de natureza psicométrica, que busca validar uma escala elaborada por meio de evidências de estrutura interna. A coleta de dados foi realizada em 18 unidades básicas de saúde que executam a metodologia da Planificação da Atenção à Saúde (PAS), distribuídas nas cinco regiões do Brasil. A versão inicial da EVO-BR continha 41 itens que mediam vulnerabilidade odontológica e foram aplicadas em usuários com 18 anos ou mais, usuários do Sistema Único de Saúde (SUS), que estivessem nas unidades básicas de saúde para consulta com profissionais de nível superior. Para avaliação das evidências, foram utilizadas as seguintes análises estatísticas: análise fatorial exploratória, análise fatorial confirmatória e *network analysis.*

**RESULTADOS:**

Participaram do estudo 1.753 usuários. Para adequação da amostra, considerou-se a fatorabilidade obtida de Kaiser-Meyer-Olkin (KMO) = 0,65, Bartlett sphericity = 8019,7 e determinante da matriz de 0,008. A análise paralela inicial indicou um modelo de quatro dimensões e teve os itens ajustados conforme cargas fatorais (variaram de 0,38 a 0,99), comunalidades (0,13 a 0,89) e Pratt’s measure, até que o modelo tivesse congruência nos princípios estatístico e interpretativo simultaneamente. O modelo final apresentou 15 itens, manteve a indicação de quatro dimensões pela análise paralela e uma variância explicada de 68,56%.

**CONCLUSÕES:**

A EVO-BR é uma escala validada para mensurar vulnerabilidade odontológica e, dessa forma, pode contribuir para organização do acesso a equipe de saúde bucal na atenção primária à saúde (APS) por meio da estratificação da população, como recomendado na planificação.

## INTRODUÇÃO

O desafio da garantia do acesso à saúde bucal de modo universal e equânime permanece no Sistema Único de Saúde (SUS). Deste modo, autores vêm discutindo formatos de organização da oferta e demanda por meio de uma gestão de base populacional, utilizando estratégias como a Planificação da Atenção à Saúde (PAS) para superar as fragmentações históricas e modelo de gestão com base na oferta^1^.

Assim, a PAS atua nos micro e macroprocessos da atenção primária à saúde (APS) e tem como base o conhecimento das subpopulações que compõe o território, de forma que o cuidado possa ser organizado considerando as suas necessidades. Para isso, são necessárias ferramentas que possam apoiar os profissionais neste processo.

A vulnerabilidade resulta de condições multidimensionais e relacionais, e é caracterizada pelo conjunto de fatores individual, social e programático, que somados levam ou não à vulnerabilidade. Na saúde bucal, recentemente, vulnerabilidade odontológica foi definida como “um conjunto de fatores sociais, estruturais, de saúde geral, mental e bucal, além de fatores envolvendo os serviços de saúde e a gestão pública que influenciam a dinâmica do processo saúde-doença em odontologia”^2^. Diante desse contexto multifatorial e da lacuna do cuidado em saúde bucal, um instrumento que permita a identificação da vulnerabilidade odontológica de forma sistemática e padronizada em todo país é fundamental para apoiar o planejamento das ações de saúde bucal de forma oportuna e equitativa.

Neste sentido, a Escala de Vulnerabilidade Odontológica (EVO) foi desenvolvida e demonstrou evidências de validade em um estudo conduzido na cidade de São Paulo, carecendo de validade em contexto nacional. Considerando a diversidade e extensão do território brasileiro, faz-se necessário investigar as evidências de validade da EVO em âmbito nacional. Assim, este estudo objetiva investigar as evidências de eficácia da estrutura interna da Escala Brasileira de Vulnerabilidade Odontológica (EVO-BR) na APS, em diferentes contextos brasileiros. Acredita-se que a partir da mensuração da vulnerabilidade odontológica, a EVO-BR possa apoiar o planejamento de políticas de saúde bucal que ampliem o acesso aos serviços de saúde, reduzindo sua inequidade no Brasil.

## MÉTODOS

Trata-se de um estudo de evidências adicionais da estrutura interna da EVO-BR, por meio da análise fatorial exploratória (AFE), confirmatória (AFC) e *network analysis* (NA), segundo recomendação atual do American Educational Research Association, American Psychological Association, e do National Council on Measurement in Education. O estudo foi aprovado pelo Comitê de Ética em Pesquisa do Hospital Israelita Albert Einstein, (CAAE: 12395919.0.0000.0071).

### Cenário do Estudo

A amostra foi composta por usuários de unidades básicas de saúde (UBS) localizadas nas cinco regiões geográficas brasileiras. A seleção das UBS foi realizada de acordo com os seguintes critérios: municípios que executam a metodologia da PAS, sendo ao menos uma UBS em cada uma das cinco regiões geográficas brasileiras; municípios mais populosos; e fácil acesso aos coletadores de dados.

A partir desses critérios, ficou definida a aplicação da EVO-BR nos municípios de: Uberlândia-MG (duas UBS) e São Paulo-SP (11 UBS) representando a região Sudeste; Irati e Teixeira Soares-PR (duas UBS) representando a região Sul, Belo Jardim-PE (uma UBS) representando a região Nordeste; Rondonópolis-MT (duas UBS) representando o Centro-Oeste; e, por fim, Boa Vista-RR representando a região Norte, com uma UBS. A coleta dos dados ocorreu em duas etapas, sendo a primeira entre setembro e novembro de 2019 (SP) e a segunda entre maio e agosto de 2022 (MG, MT, RR, PE, PR).

A primeira etapa, a coleta, foi realizada por cirurgiões-dentistas^3^ e a segunda foi realizada por coletadores treinados, durante duas semanas de visita aos municípios fora de São Paulo.

### Amostra

Foram considerados como critérios de inclusão: ser usuário do serviço; ter 18 anos de idade ou mais; e comparecer na UBS durante o período de coleta de dados. Foram excluídos os usuários com falta de informação sobre os itens da EVO (ou seja, aqueles que aceitaram participar e responderam ao instrumento de caracterização, mas tiveram a coleta de dados interrompida antes de responderem os itens previstos na escala).

Os participantes eram abordados durante o atendimento no consultório odontológico ou na sala de espera da UBS e convidados a participar da pesquisa. Após apresentação e concordância com o termo de consentimento livre e esclarecido, aplicava-se o questionário estruturado contendo uma caracterização clínica e sociodemográfica e os itens da EVO-BR (Tabela 1)^3^. O software Research Electronic Data Capture (RedCap) foi utilizado para coleta e armazenamento de dados.

**Tabela 1:** Escala de Vulnerabilidade Odontológica Inicial.

Item	Sim	Não
1. Você vive na cama?		
2. Você vive apenas dentro de casa?		
3. Você tem o vírus HIV?		
4. Você tem doença do coração?		
5. Sua saúde te impede de fazer algumas atividades diárias?		
6. Você come alimentos que possuem açúcar todos os dias?		
7. Você consome massas, batatas ou pães em todas as refeições?		
8. Você tem diabetes?		
9. Você tem alguma dificuldade de movimento?		
10. Você tem alguma doença que precisa de acompanhamento?		
11. Você tem síndrome de Down?		
12. Você fuma?		
13. Você tem câncer?		
14. Você já fez radioterapia?		
15. Você consome bebidas alcoólicas?		
16. Você faz uso de drogas?		
17. Você considera que sua família tem bons hábitos de higiene?		
18. Falta algum dente na sua boca?		
19. Você acredita que quando um dente dá problema, a melhor solução é retirá-lo?		
20. Você considera ter uma boa saúde bucal?		
21. Você tem câncer de boca?		
22. Você já teve câncer de boca?		
23. Você tem dentes cariados?		
24. Você considera importante cuidar da boca?		
25. Você acredita que doenças da boca podem ser evitadas?		
26. Você se considera responsável com a saúde da sua boca?		
27. Você considera importante ter todos os dentes na boca?		
28. Você mastiga bem?		
29. Você tem diagnóstico de depressão?		
30. Você tem acesso à água com flúor?		
31. Você tem banheiro na sua casa?		
32. Você tem luz elétrica na sua casa?		
33. Você tem água encanada em sua casa?		
34. Você tem coleta de esgoto em sua casa?		
35. Você considera sua casa distante do serviço de atendimento odontológico que utiliza?		
36. Você conhece o posto de saúde público onde pode se consultar?		
37. Você frequenta posto de saúde público?		
38. Você é acompanhado por uma equipe de saúde bucal?		
39. Você frequenta gratuitamente o dentista?		
40. Você já foi ao dentista para fazer um tratamento?		
41. Você tem plano de saúde odontológico?		

### Análise Estatística

**Análise fatorial exploratória (AFE):** a verificação dos dados é fundamental, e eles são passíveis de análise fatorial por meio da medida de adequação da amostra (*measure of sampling adequacy*). Para esta etapa, foram avaliados a Bartlett sphericity, determinante da matriz e Kaiser-Meyer-Olkin (KMO). Além da avalição do banco de dados, foi realizada análise individual dos itens conforme recomendação, dessa forma, sua inadequação para fatoração pode afetar a solução do modelo. Os valores ausentes foram tratados pela técnica de múltiplas imputações^3^.

A testagem da dimensionalidade foi realizada com a análise paralela (AP), por meio da optimal implementation of parallel analysis, com *minimun rank factor analysi*s, que minimiza a variância comum dos resíduos. A AP foi implementada por meio de matriz policórica e da permutação com 500 matrizes aleatórias, além disso, tem sido considerada uma das técnicas mais robustas e precisas para testagem de dimensionalidade^4,5^. A extração dos fatores foi feita pela técnica unweighted least squares (ULS), que reduz os resíduos das matrizes. Caso o instrumento mostre-se multidimensional, será utilizada a rotação obliqua Promin^6^. Adotou-se como indicadores de avaliação da unidimensionalidade: congruência unidimensional (Unico – unidimensional congruence) > 0,95, variância comum explicada (ECV – explained common variance) > 0,80 e média de item de cargas absolutas residuais (Mireal *–* mean of item residual absolute loadings*)* < 0,30)^7^.

**Parâmetros de qualidade dos instrumentos:** a variância explicada do instrumento deve ficar por volta de 60%^8^. As cargas fatoriais iniciais de 0,30 são recomendadas quando a amostra tem, ao menos, 300 indivíduos. A manutenção ou retirada do item no modelo dependerá da magnitude das cargas fatoriais, das comunalidades, da inexistência de dupla saturação, de casos de *Heywood* e a da interpretabilidade dos fatores. No intuito de aumentar a precisão da tomada de decisão quanto a permanência ou retirada de itens, optou-se pelo uso do unique directional correlation (ETA) pela Pratt´s measure^9^.

**Análise fatorial confirmatória (AFC):** para os dados primários da AFC, utilizou-se as cargas fatoriais e o poder preditivo do item (R^2^). Os índices de ajustamento do modelo adotados foram: χ^2^/df; índice de ajuste não-normativo (NNFI – Non-Normed Fit Index) > 0,95; índice de ajuste comparativo (CFI –Comparative Fit Index) > 0,95; índices de adequação de ajuste (GFI –Goodness Fit Index) > 0,95; raiz do erro quadrático médio de aproximação (RMSEA – Root Mean Square Error of Approximation) < 0,08; e raiz quadrática média dos resíduos (RMSR – Root Mean Square of Residuals) < 0,8. O modelo testado na AFC foi a solução fatorial encontrada no estudo inicial da AFE^2^.

A confiabilidade foi aferida por quatro indicadores: alfa de Cronbach^10^, Greatest Lower Bound ^11^, Ômega^12^ – esses três por meio de *bayesian estimation* – e Orion (Overall Reliability of Fully-Informative Prior Oblique N-EAP scores N)^13^.

**Network analysis:** na última década houve uma extensão da aplicação da *network analysis* para diversos cenários, tais como: avaliação de sintomas^14^, redes psicológicas e psicopatologias, estresse pós-traumático^15^, ansiedade^16^, desenvolvimento de instrumentos de medida^17^ e odontologia^18^.

Cabe entender como ela pode ser útil na busca por evidências de validade. Segundo Newman^19^, a análise é composta por dois estágios: no primeiro, estima um modelo estatístico de dados, a partir do qual alguns parâmetros podem ser representados como uma rede ponderada entre as variáveis observadas e, no segundo estágio, é analisada a estrutura da rede ponderada, usando medidas tiradas da teoria dos grafos.

Este estudo utilizou como estimador a técnica High-dimensional Undirected Graph Estimation (Huge)^20^ e como critério o Extended Bayesian Information Criterio (Ebic). A Huge trabalha com dois procedimentos de estimação: 1) algoritmo de busca em vizinhança; e 2) algoritmo gráfico de Lasso^21^. Os nodos dos gráficos foram posicionados utilizando o algoritmo de Fruchterman-Reingold^22^, que é baseado na força e conectividade entre os nodos, e cada nodo representa um item do instrumento.

Para avaliar o modelo gerado, foram adotados quatro indicadores: intermediação (*betweeness*), que avalia a eficiência com que um nó se conecta com os outros; proximidade (*closeness*), que avalia a facilidade com que a informação atinge os outros nós a partir de um nó específico; força (*strength or degree*), que representa o quanto um nó está conectado com o resto da rede; e, por fim, a influência esperada (*expected influence*), que visa avaliar a natureza e a força da influência cumulativa de um nó dentro da rede^23^e, portanto, o papel que se pode esperar que ele desempenhe na ativação, persistência e remissão dela^24^.

A literatura tem recomendado a adoção de múltiplas testagens e técnicas para ajustar um instrumento, atendendo as recomendações contemporâneas para evidência de validade. Essa combinação busca melhorar a precisão e a qualidade dos instrumentos^25,26^ e pode auxiliar na determinação de qual o melhor modelo quando há mais de uma possibilidade de solução^27^. Para ambas as técnicas, foi aplicada uma *bootstrap* de 5000. As análises foram efetuadas como Factor 12.02 e JASP 16.04.

## RESULTADOS

A população deste estudo compreendeu 1.753 participantes. A maioria dos respondentes era do sexo masculino (52,01%), com idade média de 39 anos (14,37%). Em relação a raça/cor, a distribuição se mostrou similar entre branco (30,20%), preto e pardo (32,69%), com predomínio de pretos (34,44%). A maioria das pessoas (81,42%) referiu que a razão moradores/cômodos do domicílio era igual ou inferior a um.

As análises foram realizadas com os 41 itens iniciais da EVO-BR (Tabela 1). A medida de adequação de amostra indicou a possibilidade de fatorabilidade com o KMO = 0,65, Bartlett sphericity = 8019,7 (df = 820; p < 0,0001) e o determinante da matriz de 0,008.

A AP inicial indicou um modelo de quatro dimensões, mas com diversos itens com níveis inadequados de carga fatorial, comunalidades e Pratt´s measure, resultando em sucessivas retiradas de itens visando o ajuste de dois princípios do modelo, estatístico e interpretativo, até que se tornassem congruentes. O modelo final com 15 itens manteve a indicação de quatro dimensões pela análise paralela e com uma variância explicada de 68,56%. Os valores de *closeness of dimensionality* indicaram um modelo multidimensional: UNICO = 0,79; ECV = 0,63 e MIREAL = 0,21. O modelo final com as cargas fatoriais, comunalidade e Pratt´s measure pode ser visto na Tabela 2. As cargas fatoriais do instrumento variaram de 0,38 a 0,99, comunalidades de 0,13 a 0,89 e o ETA do Pratt’s measure de 0,36 a 0,94

**Tabela 2:** Cargas fatoriais, comunalidades e Pratt´s measure.

Item		Cargas fatoriais				h^2^	Pratt´s measure (ETA)			
	
		Saúde geral	Saúde oral	Infraestrutura	Serviços de saúde		Saúde geral	Saúde oral	Infraestrutura	Serviços de saúde
V1	Sua saúde te impede de fazer algumas atividades diárias?	**0,946**	0,011	-0,003	0,000	0,894	**0,945**	0,011	0,000	0,000
V2	Você tem alguma dificuldade de movimento?	**0,831**	-0,049	-0,002	0,044	0,696	**0,832**	0,046	0,000	0,053
V3	Você tem alguma doença que precisa de acompanhamento?	**0,680**	0,068	-0,064	-0,037	0,466	**0,678**	0,054	0,045	0,029
V4	Você considera importante cuidar da boca?	-0,061	**0,548**	-0,012	0,179	0,356	0,057	**0,556**	0,000	0,209
V5	Você acredita que doenças da boca podem ser evitadas?	0,069	**0,467**	0,082	0,034	0,262	0,071	**0,483**	0,143	0,064
V6	Você se considera responsável com a saúde da sua boca?	-0,118	**0,522**	0,005	0,087	0,307	0,116	**0,528**	0,033	0,115
V7	Você considera importante ter todos os dentes na boca?	0,019	**0,993**	-0,011	-0,073	0,965	0,018	**0,982**	0,000	0,000
V8	Você tem banheiro na sua casa?	-0,079	0,201	**0,857**	0,138	0,982	0,063	0,319	**0,907**	0,231
V9	Você tem luz elétrica na sua casa?	-0,034	-0,007	**0,647**	0,002	0,417	0,023	0	**0,645**	0,020
V10	Você tem água encanada em sua casa?	0,044	-0,043	**0,932**	0,042	0,87	0,056	0	**0,925**	0,109
V11	Você tem coleta de esgoto em sua casa?	0,009	-0,127	**0,861**	-0,164	0,643	0,016	0	**0,801**	0,000
V12	Você conhece o posto de saúde público onde pode se consultar?	0,039	0,211	0,221	**0,510**	0,475	0,049	0,273	0,307	**0,552**
V13	Você frequenta posto de saúde público?	0,136	0,07	0,179	**0,761**	0,737	0,151	0,127	0,269	**0,791**
V14	Você é acompanhado por uma equipe de saúde bucal?	-0,054	-0,01	-0,062	**0,382**	0,139	0,048	0,000	0,000	**0,369**
V15	Você frequenta gratuitamente o dentista?	-0,079	-0,142	-0,13	**0,831**	0,655	0,066	0,104	0,000	**0,800**

**Negrito** – cargas fatoriais substantivas (> 0.30).

ETA: Unique Directional Correlation.

A dimensão 1 foi denominada de “Saúde geral” e composta pelos itens de 1 a 3, a dimensão 2 de “Saúde oral” pelos itens de 4 a 7, a terceira dimensão foi denominada “Infraestrutura” com os itens de 8 a 11 e a última foi a “Serviços de saúde” com os itens de 12 a 15. O modelo ficou idêntico ao modelo inicial^3^.

Os índices de confiabilidade com estimação bayesiana foram de alfa de Cronbach = 0,81 (IC95% 0,79–0,82), ômega de McDonald = 0,78 (IC95% 0,76–0,81) e GLB = 0,93 (IC95% 0,92–0,93). O Orion foi, respectivamente, para cada dimensão, de 0,95, 0,84, 0,92 e 0,98. Todos os indicadores estão em níveis adequados.

O modelo obtido na AFE foi replicado na AFC, as cargas fatoriais variaram de 0,20 a 0,96 e a capacidade preditiva do item (R2) de 0,09 a 0,92 (Figura 1). A “Saúde geral” teve cargas fatoriais oscilando entre 0,48 e 0,78; para “Saúde oral” de 0,28 a 0,54; para “Infraestrutura” foram de 0,34 a 0,96 e para “Serviços de saúde” foram entre 0,20 e 0,76. Além dos indicadores primários, os índices de qualidade do modelo estabeleceram-se em: X^2^_(51)_ = 2,96, p < 0,001; NNFI = 0,98, CFI = 0,99, GFI = 0,98, RMSEA = 0,03 e RMSR = 0,04. Os indicadores estabeleceram-se em níveis satisfatórios e consistentes.

**Figura 1:** Diagrama de caminho.

A *network analysis* foi aplicada ao modelo estabelecido anteriormente (Figura 2). A Figura 3 mostra que os itens se associaram de forma adequada a seus pares, respeitando os domínios estabelecidos. O que indica, novamente, a sustentabilidade e estabilidade do modelo proposto.

**Figura 2:** Network analysis da EVO-BR.

EVO-BR: Escala Brasileira de Vulnerabilidade Odontológica.

**Figura 3:** Índices de centralidade dos itens normatizados (z).

Para os indicadores de centralidade normatizados, os itens (Figura 3) que apresentaram resultados mais relevantes foram: para a intermediação (*betweeness*), os itens 3 “Você tem alguma doença que precisa de acompanhamento?” e 8 “Você tem banheiro na sua casa?”. Para a proximidade (*closeness*), os itens 3 “Você tem alguma doença que precisa de acompanhamento?”, 4 “Você considera importante cuidar da boca?”, 6 “Você se considera responsável com a saúde da sua boca?”, 7 “Você considera importante ter todos os dentes na boca?” e 8 “Você tem banheiro na sua casa?”, portanto, aqueles que permitem o fluxo de informação num espaço mais curto. Para o índice força/grau (*strenght/degree*) e influência esperada, o item 7 “Você considera importante ter todos os dentes na boca?” apresentou o melhor resultado, indicando que tem a maior força de conexão e a maior influência cumulativa para a configuração do modelo.

## DISCUSSÃO

Este estudo demonstrou, por meio do modelo inicial na AFE com uma amostra multicêntrica e da replicação desse modelo na AFC e na *network analysis*, que a EVO-BR apresenta consistência, robustez e confiabilidade no cenário brasileiro. Além das propriedades psicométricas adequadas, a versão final da escala é um instrumento prático, conciso e de fácil aplicação, uma vez que é composta por apenas 15 itens, todos com opções de respostas não ou sim. Desta forma, a EVO-BR minimiza a subjetividade das respostas e permite a mensuração da vulnerabilidade odontológica de forma assertiva por diferentes profissionais da APS, incluindo o agente comunitário de saúde, para além dos profissionais dentistas.

Apesar deste estudo ter sido conduzido com amostra de conveniência, não representativa da população brasileira do ponto de vista estatístico, destaca-se como ponto forte o fato de ter considerado usuários das cinco regiões geográficas brasileiras, incorporando a diversidade territorial, cultural e social do país.

Corroborando o estudo conduzido em São Paulo^3^, a EVO-BR mantém as dimensões de saúde geral, saúde oral, infraestrutura e serviços de saúde, para possibilitar a mensuração da vulnerabilidade odontológica. A saúde geral e bucal apresentam forte correlação quando se fala em processo saúde-doença e impactam na qualidade de vida do paciente, tornando-se indissociáveis^28^. Esse fato pode ser explicado pela associação dos problemas bucais com desequilíbrios nutricionais, interferência na qualidade do sono, fonação, agravamento de condições psicossociais, entre outros.

Segundo Gonçalves et al.^29^, a maioria das UBS no Brasil não apresentam infraestrutura necessária para garantir acesso universal e acessibilidade aos serviços odontológicos, o que ressalta a importância desta dimensão para pelo menos 23,1% dos brasileiros que possuem algum tipo de limitação^29^. Uma infraestrutura de serviços de saúde bucal ruim está associada a uma menor utilização dos serviços odontológicos, sendo considerados fundamentais para caracterização da vulnerabilidade odontológica.

Fatores históricos inerentes a entrada tardia da odontologia na estratégia saúde da família contribuiu para um menor cuidado da população com saúde bucal, bem como para uma gestão fragilizada. Estima-se que cerca de 13% da população brasileira nunca tenha ido ao dentista^30^. Os itens da EVO-BR, na dimensão saúde oral, contemplam justamente esse olhar de autopercepção que traz os resultados das experiências e qualidade de vida do indivíduo, retratando suas necessidades, o que corrobora o modelo de gestão populacional e confronta o modelo assistencial de práticas mutiladoras e baixa resolutividade vigente por anos.

Sabe-se que indivíduos com melhores condições de vida apresentam maior possibilidade de receber assistência odontológica^28^. Neste contexto, a dimensão de serviços de saúde considera o acesso e a utilização dos serviços na mensuração da vulnerabilidade odontológica.

A metodologia da PAS tem como objetivo apoiar gestores e profissionais de saúde na organização dos serviços e redes de atenção à saúde, utilizando como referencial o Modelo de Atenção às Condições Crônicas (Macc)^1^. O Macc considera elementos do modelo de atenção crônica, do modelo da pirâmide de risco e do modelo da determinação social da saúde. Assim, sugere-se que os processos de trabalho sejam organizados a partir do conhecimento de fatores sociais, para além dos fatores de risco biopsicossociais^1^.

## CONCLUSÃO

A EVO-BR mostrou-se como potencial instrumento para ser utilizado na PAS, de modo a apoiar o processo de trabalho e o planejamento das ações de saúde bucal na APS a partir das necessidades dos usuários e, assim, favorecendo o cuidado integral, equitativo e oportuno aos usuários. Espera-se que, a partir da identificação dos vulneráveis, a EVO-BR represente uma estratégia que contribua para avançarmos na discussão de organização da oferta de saúde bucal no Brasil.
